# The spatial distribution characteristics of typical pathogens and nitrogen and phosphorus in the sediments of Shahe Reservoir and their relationships

**DOI:** 10.1038/s41598-021-01252-z

**Published:** 2021-11-05

**Authors:** Wen Sun, Ke Yang, Risheng Li, Tianqing Chen, Longfei Xia, Zhao Wang, Xubo Sun

**Affiliations:** 1 Shaanxi Provincial Land Engineering Construction Group, Key Laboratory of Degraded and Unused Land Consolidation Engineering, Ministry of Natural Resources, Xian, 710075 China; 2 Shaanxi Provincial Land Engineering Construction Group, Shaanxi Provincial Land Consolidation Engineering Technology Research Center, Xian, 710075 China; 3 Shaanxi Provincial Land Engineering Construction Group, Land Engineering Technology Innovation Center, Ministry of Natural Resources, Xian, 710075 China; 4 Land Engineering Quality Testing of Shaanxi Land Engineering Construction Group Co., Ltd, Xian, 710075 China

**Keywords:** Pollution remediation, Environmental impact

## Abstract

Using samples collected in Shahe Reservoir in the upper North Canal in China, this research analyzes the structure of a microorganism group in sediment and the absolute abundance of two typical pathogenic bacteria (*Escherichia coli* and *Enterococcus*), and their relationship with environmental factors including total nitrogen (TN) and total phosphorus (TP). The study of samples collected from the surface (0–20 cm) and sediment cores shows that the absolute abundance of *E. coli* in horizontal distribution in the sediment is highest in downstream of the reservoir and point source pollution area. In vertical distribution, the absolute gene expression level of the two pathogenic bacteria in the sediment tends to decrease with increasing depth, although its highest value at 10–30 cm depth. The relative abundance the two pathogenic bacteria is much greater in the sediment of Shahe Reservoir with the structure of horizontal groups including *Clortridium *sensu stricto*,* unclassified *Anaeroineaceae*, and *Povalibacter*, while *Anaeroineaceae* is much more abundant in the group structure of the vertical distribution. Pearson correlation analysis suggests positive correlation in horizontal distribution for *E. coli* and TN and TP (*P* < 0.05) and for *Enterococcus* and TP (*P* < 0.05). The results clearly show that the amount of pathogenic bacteria in the sediment in Shahe Reservoir is most likely due to water eutrophication.

## Introduction

The contamination of surface water bodies by pathogenic bacteria poses a huge potential threat to human health. The seven major water systems in China have all been contaminated by pathogenic bacteria to varying degrees^1^. Pathogens are widely distributed in both surface water and sediments, while the sediments themselves can provide various protections for pathogens (reducing ultraviolet radiation, providing nutrients, etc.)^2^. Thus, sediments can aid the long-term survival and growth of pathogenic bacteria in water bodies, acting as both the “source” and “sink”^3^. Studies have shown that those pathogenic bacteria that are widely enriched in surface water sediments include *Escherichia coli* (*E. coli*), fecal coliform (*FC*), *Enterococcus* (*ENT*), total coliform (*TC*), *Campylobacter* and *Salmonella*, etc., and that disturbances to the sediment or water can cause the further release and adsorption of pathogenic bacteria^4–7^. Some scholars have reported on the investigation of microbial diversity in the water system of the North Canal and the evaluation of river health. For example, Wang Mi et al.^8^ investigated the microorganisms of the North Canal water system, and the results of water quality evaluation based on the microorganisms showed that the North Canal water system was in a state of moderate pollution; Gu Xiaoyun et al.^9^ developed the ecosystem of the North Canal (Beijing section) The health assessment pointed out that the health status of the North Canal ecosystem is generally poor. In addition, a large number of studies have shown that nitrogen and phosphorus in sediments are fundamental and crucial factors affecting the eutrophication of water bodies. However, the relationship between pathogenic bacteria and nitrogen and phosphorus in surface water sediments is not yet clear.

The current strict implementation of the Water Pollution Prevention and Control Action Plan in China has focused more on organic pollution and eutrophication, but the prevention and control of pathogenic microorganism pollution in rivers need to be further strengthened^10^ to be more conducive to public health safety management of river basins^11^. For example, in addition to the problem of eutrophication in the Wenyu section of the North Canal, the upstream and downstream microbic pollution is severe, with the surface water concentration of *FC* on average exceeding the Class V water quality standard (GB3838-2002) by two orders of magnitude. At present, related researches mostly focus on the relationship between nitrogen and phosphorus nutrients and algae or the relationship between microbes and algae. However, there are few studies on the relationship between nitrogen and phosphorus nutrients and pathogenic microorganisms. Therefore, this study chose Shahe Reservoir in the upper reaches of the North Canal as the research site to investigate the spatial distribution characteristics of microbic communities and nitrogen and phosphorus in sediments, and selected the characteristic pathogenic bacteria *E. coli* and *ENT* for this analysis. The relationships between these typical pathogenic bacteria and nitrogen and phosphorus are expected to provide a scientific basis for the treatment of river pathogen pollution and eutrophication.

## Materials and methods

### Overview of the study area

**Figure 1:**
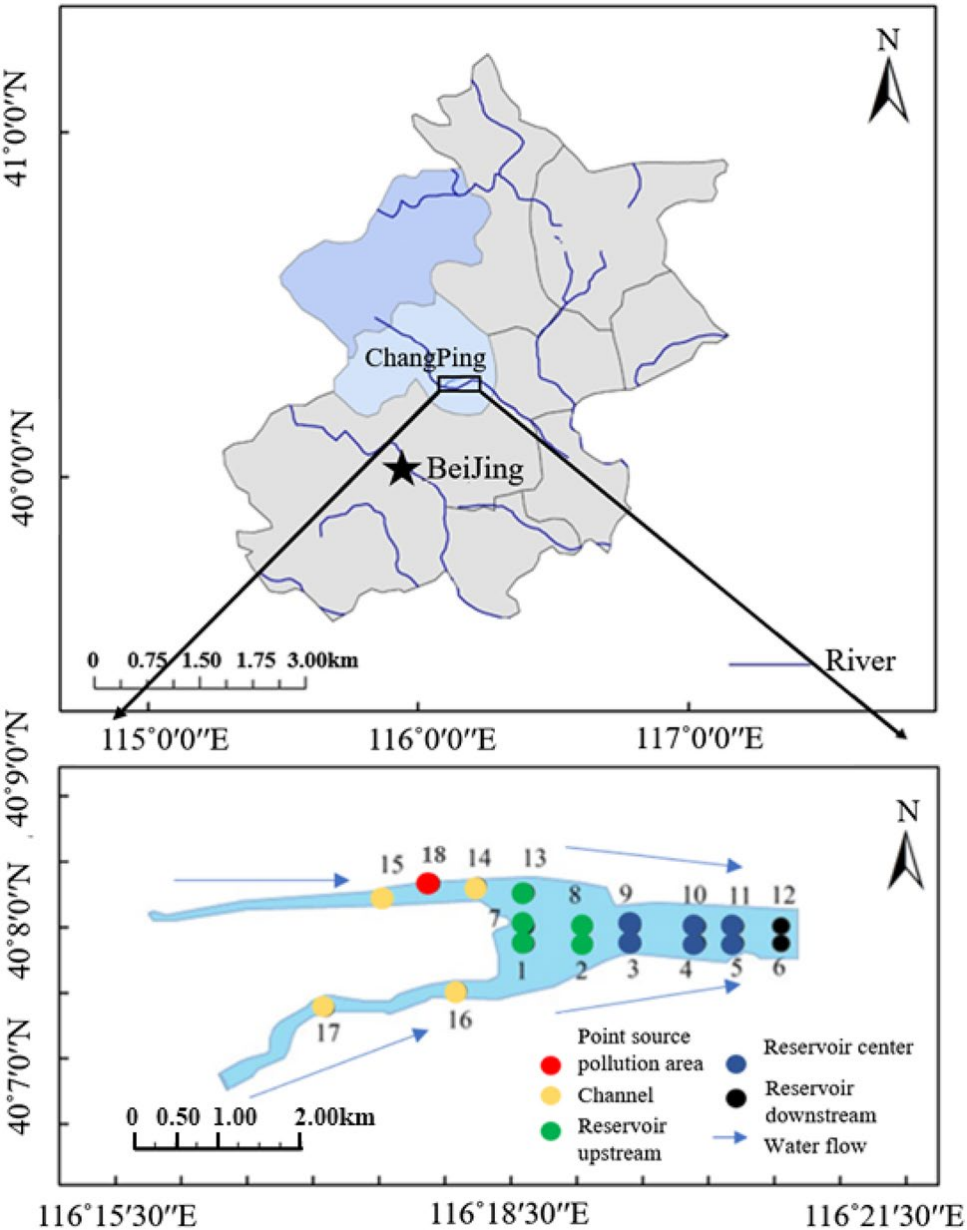
Arrangement and zoning map of sediment and interstitial water sampling points at Shahe Reservoir. (The figure was created by Sun Wen^16^ and modified using ArcGIS software 10.2; Source:WGS 1984).

Shahe Reservoir is an important node located in the source area of the North Canal (Fig. 1). The drainage area of Shahe Reservoir is about 1125 km^2^, of which the mountain area accounts for about 75% ^12^. The North Canal is an important drainage channel for Beijing. From 1999 to 2005, the average annual sewage storage volume accounted for about 55% of the total incoming water^13^. The three main tributaries that merge into the Shahe Reservoir, Beisha River, Dongsha River, and Nansha River, have drainage areas of 597 km^2^, 265 km^2^, and 263 km^2^, respectively^14^. The Shahe Reservoir is a river-type reservoir controlled by the Shahe Sluice, and was built in 1960. Total area around the reservoir is about 1.8 km^2^. The annual average water level is about 36 m, total storage capacity is 20.45 million m^3^, and the historical daily mean outflow is about 125,000 m^3^ per day. The hydraulic retention time is 69–110 days, and the fluidity is poor during the water storage period. During the annual flood season (June–September), the Shahe Reservoir will open the gate and release the water. At this time, the water level will drop rapidly and the flow velocity will increase significantly^15^.

### Sample collection and processing

Based on their topographical characteristics, 18 sediment sampling points were set up within the Shahe Reservoir study area (Fig. 1). In November 2017, a Peterson mud harvester was used to collect 0–20 cm surface sediment to analyze the horizontal distribution characteristics of nutrients and pathogens. Three columnar sediment samples were collected at sampling points 3^#^, 14^#^, and 16^#^ using a mud core sampler to analyze the vertical distribution characteristics of nutrients and pathogens. (r = 50 mm, h = 60 cm).

The collected surface sediment was protected from light, stored at low temperature, and then brought back to the laboratory. The sediment columns were layered at 2 cm intervals, and the layered samples and sediment surface samples were freeze-dried (Model FD-1A-50 freeze dryer, Beijing Boyikang Experimental Instrument Co., Ltd.), crushed with a glass rod to remove impurities such as gravel, shells, and animal and plant residues, ground with a mortar, and passed through a 100-mesh sieve before analysis. Meanwhile, the samples obtained by the Peterson mud harvester were mixed and put into a 50 mL centrifuge tube, centrifuged at 4000 rpm for 20 min to obtain interstitial water, and stored at − 4 °C.

Take a fresh sample of the sediment and use the drying method to determine the moisture content and organic matter (expressed as loss on ignition, LOI). An elemental analyzer (Vario MAX cube, Elementar) was used to determine the total nitrogen (TN) content value in the sediment. Total Phosphorus (TP) in the sediment was extracted by the SMT (Standards, Measurements and Testing) method developed under the framework of the European Standards and Testing Committee. The sediment samples were burned at 450 °C and oscillated at room temperature with 3.5 mol/L HCl After 16 h, the molybdenum antimony spectrophotometric method was used to determine the TP content in the extract.

In this study, the typical pathogenic bacteria Escherichia coli and Enterococcus were selected as indicator bacteria for quantitative analysis of qPCR, and the gene copy number (DNA copies·g^−1^) was used to express its corresponding content in the sediment, and the relative abundance was standardized by 16S rRNA.

#### DNA extraction

the sediment sample is freeze-dried and weighed 0.1 g In a 2 mL lysis tube, use the FastDNA Spin Kit for Soil kit (MPbio, USA), and extract DNA according to the instructions of the kit.

#### Microbial community structure analysis

Based on high-throughput sequencing to determine the 16S rRNA V4 region PCR product gene sequence, and analyze the microbial community structure in each sample. The PCR primer used is 515F/806R, and the barcode sequence is added before the forward primer to distinguish the PCR products of different samples. Each sample is repeated 3 times for PCR and mixed, and then the PCR products are recovered from different samples. The PCR products were mixed in equal amounts, library construction and sequencing; library construction and sequencing were completed by Sangong Bioengineering (Shanghai) Co., Ltd. The sequencing platform was Illumina MiseqTM. For Miseq paired-end sequencing data, first remove the primer linker sequence (TGGAATTTCTCTGGGTGCCCAAGGAACTC), and then according to the overlap relationship between PE reads, the paired reads are spliced ​​into a sequence and distinguished according to each sequence of the samples, and then the samples are distinguished according to the sequence. Sample data, and finally perform quality control filtering on the quality of each sample data to obtain valid data for each sample.

#### Quantitative PCR (qPCR) analysis

The main reagents used for quantitative PCR (qPCR) analysis in this study were SYBR® Premix Ex Taq™ (Tli RNaseH Plus) (TAKARA) and RNase-free Water (Ambion). The qPCR analysis was carried out on a micro ultraviolet spectrophotometer (Nanodrop 2000) and a fluorescent quantitative PCR instrument (StepOne Plus). The amplification efficiencies of the target gene fragments of *Enterococcus* and *E. coli* were 99.54% and 97.82%, respectively. The specific primer sequences and mechanisms are shown in Table 1.

**Table 1:** Primers and their mechanisms used in this study.

Target genes	Primer	Sequences	Amplico Size (bp)	Annealing Temp (℃)
16 s rRNA	1369F	CGGTGAATACGTTCYCGG	128	55
	1492R	GGWTACCTTGTTACGACTT		
*Enterococci*	ECST784F	AGAAATTCCAAACGAACTTG	93	55
	ENC854R	CAGTGCTCTACCTCCATCATT		
*E. coli*	23 s rRNA-F	GGT AGA GCA CTG TTT TGG CA	87	60
	23 s rRNA-R	TGT CTC CCG TGA TAA CTT TCTC		

### Analysis of microbial community structure and typical pathogens

Based on metagenomic classification and sequencing, the PCR products of 16S rRNA V4 regions were determined, and the microbial community structure in each sample was analyzed. The PCR primer used was 515F/806R, and the barcode sequence was added before the forward primer to distinguish the PCR products of different samples. The PCR for each sample was repeated three times before they were mixed. For this, the PCR products were recovered using gel, and the PCR products from different samples were mixed in equal amounts for library construction and sequencing; library construction and sequencing were completed by related sequencing companies, and the sequencing platform was Illumina Miseq × 250. For Miseq paired-end sequencing data, the primer adapter sequence (TGGAATTCTCGGGTGCCAAGGAACTC) needed to be removed first, and then the paired reads were merged into a sequence according to the overlap relationship between paired-end reads. Samples were then identified and distinguished according to the barcode tag sequence. Finally, quality control filtering was performed on the samples to ensure valid data for each sample.

Following guidance on relevant standards for pathogens in surface waters from the United States Environmental Protection Agency, the European Union, and the World Health Organization, this study selected the typical pathogens *E. coli* and *ENT* for analysis, using their gene copy numbers (copies g^−1^ ) to represent their corresponding content in the sediment, and their proportion (%) in 16S rRNA to represent their abundance.

### Data processing and analysis methods

SPSS 25.0 software was used to analyze the correlation between the pathogens and total nitrogen (TN) and total phosphorus (TP) in sediments. The horizontal spatial distribution characteristics of nutrients in the sediments were analyzed using the ArcGIS 10.2 software package, The projection coordinate system was selected as "Chinese Albers Projection", and the analysis method was selected as the inverse distance weighting interpolation method. The vertical spatial distribution of nutrients in the sediment and the absolute content of pathogens (copies g^−1^) were analyzed by Origin 2017. The heat map of the microbial community structure in the sediment was constructed using Heml 1.0 (http://hemi.biocuckoo.org/down.php) (Wankun et al., 2014). The R language ade4 package was used to perform noise reduction analysis on operational taxonomic units (OTUs) in the community structure. The average abundance of OTUs in all samples was required to be higher than 0.01%. The OTUs after noise reduction analysis were used for subsequent analysis.

## Results and discussion

### Spatial distribution characteristics of typical pathogens in sediments

#### Analysis of microbial community structure in surface sediments

**Figure 2:**
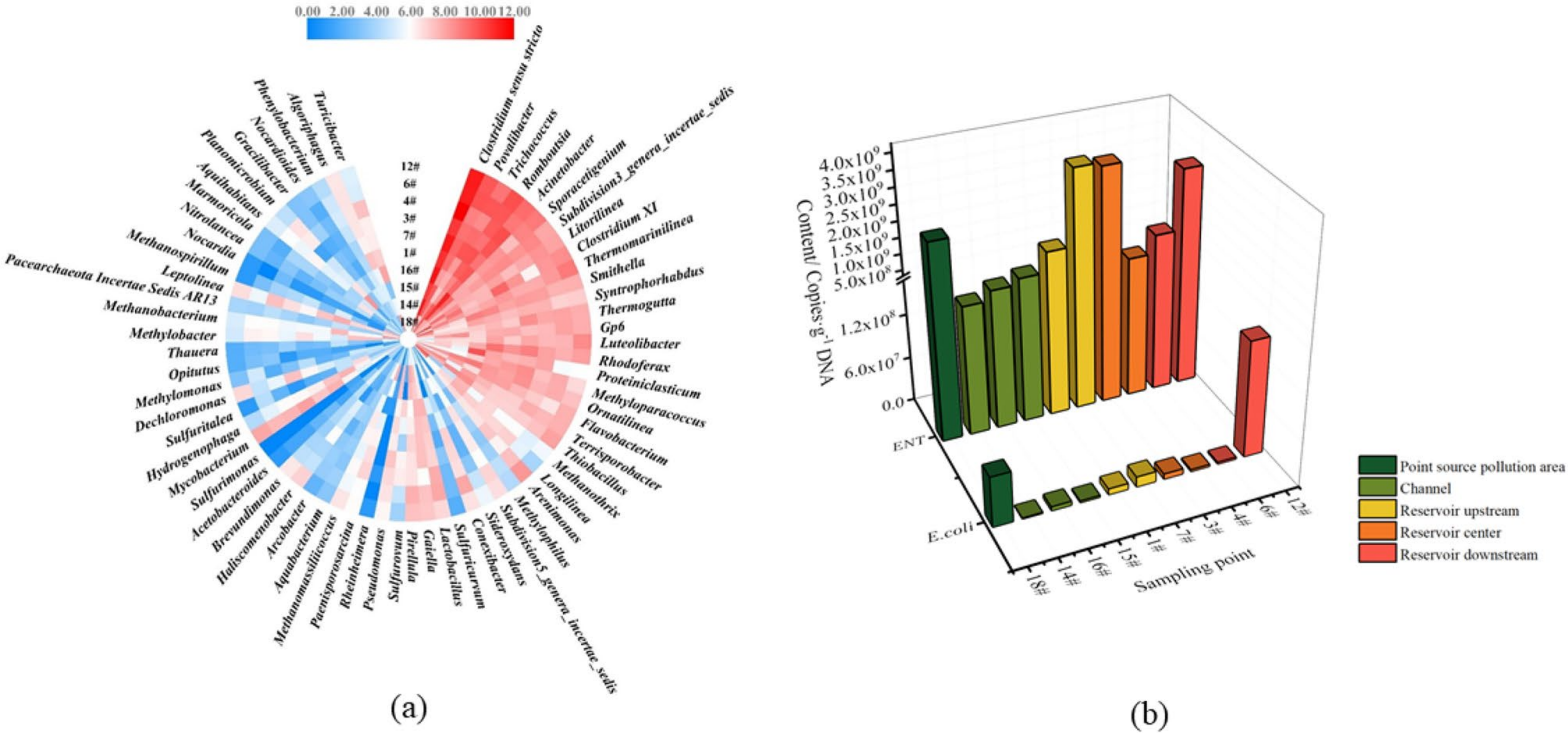
(**a**) Heat map of the top 10 genera in each sample based on the reads (log2 transformed). (**b**) Horizontal distribution of *E. coli* and *Enterococcus* in Shahe Reservoir sediments. (The Fig. 3a has been prepared using Heml 1.0 software).

It can be seen from Fig. 2 that the microbial community structures of the various surface sediments of Shahe Reservoir contained a large number of potential pathogens that had certain similarities and differences. *Clostridium *sensu stricto, a potential pathogen which is widely distributed in soil, sludge, human and animal intestines, etc., had the greatest average abundance in each sediment sample (18.97 ± 5.80%); it peaked in the central area (4^#^) at 30.03%, and its abundance in the point-source pollution area (18^#^) was lowest at 10.15%. The high abundance of *Clostridium *sensu stricto means that the sediments in the Shahe Reservoir are in an anaerobic environment. *Acinetobacter* is a pathogen widely distributed in soil and water. Its average abundance in each sediment sample was 7.90 ± 4.51%. The Beisha River channel (15^#^) had the highest abundance (18.39%). The abundance of the point-source pollution area (18^#^) was the lowest at 2.26%.

*Romboutsia*, a common intestinal pathogen, had a high average abundance in the sediments of Shahe Reservoir (6.55 ± 2.00%), but its distribution was completely opposite to the distribution of *Acinetobacter*. *Romboutsia* had its highest abundance (9.40%) in the point-source pollution area (18^#^) and its lowest abundance (2.87%) in the river channel (15^#^).

The average abundance of *Povalibacter* and *Trichococcus* species reached 5.34 ± 2.24% and 3.94 ± 2.22%, respectively, but they also showed low abundance at the point-source pollution area (18^#^), whereas abundance at sampling points in other areas was high. The highest abundance for *Povalibacter* and *Trichococcus* was 8.16% (downstream of the reservoir, 6^#^) and 7.71% (upstream of the reservoir, 7^#^), respectively, and the lowest was 0.93% (point-source pollution area, 18^#^) and 1.25% (channel, 15^#^ sampling point).

The average abundances of *Sporacetigenium*, *Subdivision3_genera_incertae_sedis*, *Clostridium XI*, *Litorilinea*, *Smithella*, and *Thermomarinilinea* were all above 2.00%: their maximum values were 5.99% (15^#^), 3.50% (3^#^), 13.06% (18^#^), 5.25% (14^#^), 7.56% (18^#^), and 4.22% (12^#^), respectively, while the minimum values were1.43% (4^#^), 1.46% (14^#^), 0.59%(4^#^), 1.54% (3^#^), 0.97% (15^#^), and 0.83% (18^#^). Thus, different microorganisms showed certain individual differences, but generally they had relatively low population abundance in the reservoir core area and point-source pollution area, and displayed high population abundance in different regions of Shahe Reservoir.

#### Horizontal distribution characteristics of E. coli and Enterococcus in surface sediments

The results of this study (Fig. 2) showed significant differences in the distribution of *E. coli* and *Enterococcus* in different regions. The horizontal distribution range of *E. coli* content was between 1.50 × 10^6^ and 1.56 × 10^8^ copies·g^−1^, with an average value of 2.69 × 10^7^ ± 4.71 × 10^7^ copies·g^−1^. The highest value was at the 12^#^ sampling point in the lower reaches of Shahe Reservoir (1.56 × 10^8^ copies·g^−1^), which was one to two orders of magnitude greater than at other regions. The average content of *E. coli* in the downstream area (6^#^, 12^#^) reached 7.97 × 10^7^ ± 7.68 × 10^7^ copies·g^−1^, followed by the point-source pollution areas (18^#^, 6.76 × 10^7^ copies·g^−1^), which were higher than the central area (3^#^, 4^#^, 5.15 × 10^6^ ± 2.26 × 10^6^ copies·g^−1^) and upstream (1^#^, 7^#^, 1.11 × 10^7^ ± 2.84 × 10^6^ copies·g^−1^), while the average content in the river channel (14^#^, 15^#^, 16^#^) was relatively low (3.26 × 10^6^ ± 1.75 × 10^6^ copies·g^−1^).

The horizontal distribution range of *ENT* content was between 3.56 × 10^8^ and 3.74 × 10^9^ copies·g^−1^, with an average value of 1.82 × 10^9^ ± 1.23 × 10^9^ copies·g^−1^. The highest value appeared in the upper reaches of Shahe Reservoir at the 7# sampling point (3.74 × 10^9^ copies·g^−1^) and was about an order of magnitude higher than at the sampling points in other regions. The average content of *ENT* in the central area (2.60 × 10^9^ ± 1.15 × 10^9^ copies·g^−1^) was relatively high, followed by the point-source pollution area (2.46 × 10^9^ copies·g^−1^), downstream (2.17 × 10^9^ ± 9.19 × 10^8^ copies·g^−1^), upstream (2.17 × 10^9^ ± 1.47 × 10^9^ copies·g^−1^), and finally in the Shahe Reservoir (6.13 × 10^8^ ± 1.96 × 10^8^ copies·g^−1^), where the content was relatively low.

The above results show that the content of *ENT* in the surface sediments of Shahe Reservoir was about two orders of magnitude higher than that of *E. coli*. Further, the content of *E. coli* in the 12^#^ sampling point downstream of the reservoir and the content of *ENT* in the surface sediments of the upstream area of ​​the reservoir are higher than in other areas.

#### Vertical distribution characteristics of microbial community structure in sediments

**Figure 3:**
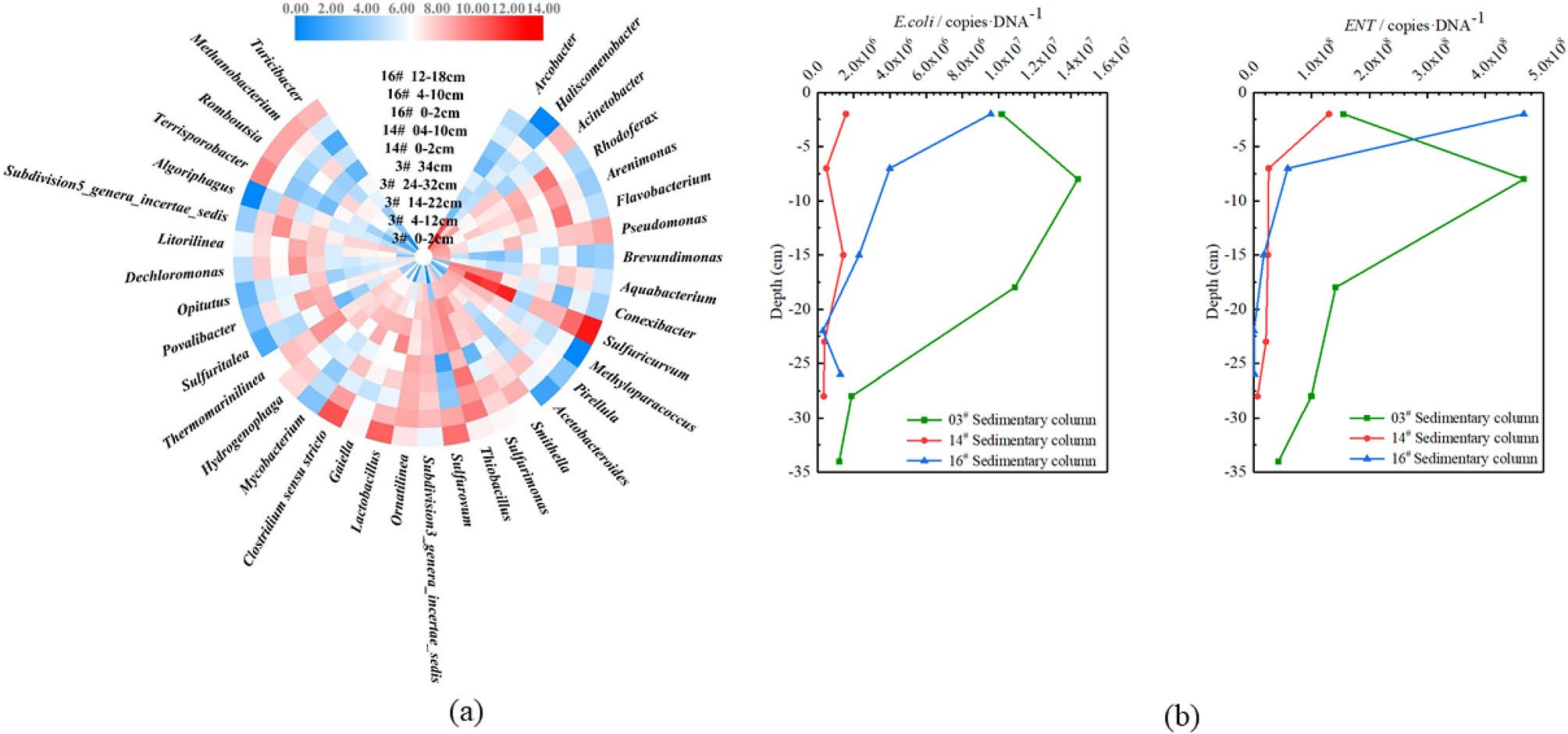
(**a**) Heat map of the top 10 genera in each sample based on the reads (log2 transformed). (**b**) Vertical distribution of *E. coli* and *Enterococcus* in Shahe Reservoir sediments. (The Fig. 3a has been prepared using Heml 1.0 software).

Based on the results of OTU classification to the genus level, a heat map (Fig. 3a) was constructed to study the vertical distribution characteristics of the microbial community structure in the sediments of Shahe Reservoir. A large number of potential pathogenic bacteria were found, which displayed certain commonalities (between different depths of the same sediment column) as well as differences (between different sediment columns).

*Sulfuricurvum,* a genus of potential pathogens that is widely distributed and commonly found in soil and sludge, had the highest average abundance (18.77% ± 16.71%) in the sediment column samples. Among these, in the 3# sediment column from the reservoir center it was most abundant (44.52%) at the depth of 34 cm, but at its lowest (0.56%) at 2 cm from the surface.

*Arcobacter* is a genus of pathogenic bacteria commonly found in humans, animals, and the environment. *A. cryaerophilus* can cause inflammation of the human intestines. The symptoms of *A. butzleri* infection include abdominal pain, nausea, vomiting, and diarrhea caused by fever. The average abundance of *Arcobacter* found in each sediment column sample was relatively high (8.35% ± 18.09%). However, contrary to the vertical distribution of *Sulfuricurvum* in the 3^#^ sediment column from Shahe Reservoir center, *Arcobacter* had its highest abundance (59.13%) at 2 cm. The abundance was lowest (0.07%) at 14–22 cm of the 3^#^ sediment column.

*Thiobacillus* had a relatively high abundance in the vertical distribution of sediments in Shahe Reservoir (6.59% ± 4.66%). The average abundance of *Clostridium *sensu stricto, *Lactobacillus*, and *Conexibacter* in the vertical distribution of the sedimentary column were above 3.00%, with the highest abundance values ​​of *Clostridium *sensu stricto and *Lactobacillus* appearing in the Nansha River channel 16^#^ sediment column at 12–18 cm (respectively, 13.75%, 10.70%), and the highest value of *Conexibacter* abundance at 4–12 cm (10.41) of the 3^#^ sediment column from the reservoir center. The minimum values ​​of the three species were in the surface 2 cm of the 3^#^ sediment column (0.56%, 0.26%, 0.06%).

#### Vertical distribution characteristics of E. coli and Enterococcus in sediments

The vertical distributions of *E. coli* and *ENT* in the sediments of Shahe Reservoir are shown in Fig. 3b. The content of *E. coli* in the 3^#^ sediment column from the reservoir center, the 14^#^ sediment column from Beisha River, and the 16# sediment column from Nansha River ranged, respectively, between 1.76 × 10^−4^ and 2.95 × 10^−4^ copies·g^−1^, 1.04 × 10^−3^ and 2.97 × 10^−2^ copies·g^−1^, and 3.34 × 10^−4^ and 6.56 × 10^−2^ copies·g^−1^. The mean values of each were 2.25 × 10^−4^ ± 4.19 × 10^−5^ copies·g^−1^, 1.55 × 10^−2^ ± 1.19 × 10^−2^ copies·g^−1^ and 2.48 × 10^−2^ ± 2.89 × 10^−2^ copies·g^−1^. Thus, it was found that the content of *E. coli* in the 16^#^ column from the Nansha River channel was about 1.6 times that of the 14^#^ column from Beisha River channel, and two orders of magnitude higher than that of the 3^#^ column from the reservoir center. It is worth noting that the *E. coli* in all three sediment columns showed a gradual increasing trend with the increase of depth. The content of the three columns at 2 cm from the surface was relatively low (1.76 × 10^−4^, 1.53 × 10^−3^ and 3.34 × 10^−4^ copies·g^−1^), but became higher at about 15–25 cm (2.10 × 10^−4^, 2.97 × 10^−2^ and 6.56 × 10^−2^ copies·g^−1^).

There was little difference between the vertical distributions of *ENT* and *E. coli*. The content of *ENT* in the 3^#^column, 14^#^column, and 16^#^column ranged, respectively, between 2.44 × 10^−3^ and 2.13 × 10^−2^ copies·g^−1^, 1.48 × 10^−2^ and 1.14 × 10^−1^ copies·g^−1^, and 1.32 × 10^−2^ and 5.12 × 10^−2^ copies·g^−1^. The average values were, respectively, 1.38 × 10^−2^ ± 6.73 × 10^−3^ copies·g^−1^, 6.15 × 10^−2^ ± 4.00 × 10^−2^ copies·g^−1^, and 2.99 × 10^−2^ ± 1.53 × 10^−2^ copies·g^−1^. Unlike *E. coli*, the content of *ENT* in the Beisha River 14^#^ sediment column was relatively high, about 2.06 times that of the Nansha River 16# column and 4.45 times that of the reservoir center 3^#^ column. The vertical distribution was the same as for *E. coli*. The content of *ENT* in the 3^#^ column from the reservoir center, the 14^#^ Beisha River column, and the 16^#^ Nansha River column were all lower at 2 cm from the surface of the sediment (respectively, 2.44 × 10^−3^ copies·g^−1^, 1.48 × 10^−2^ copies·g^−1^ and 1.32 × 10^−2^ copies·g^−1^), and higher at about 15–25 cm (2.13 × 10^−2^ copies·g^− 1^, 1.14 × 10^−1^ copies·g^−1^ and 4.44 × 10^−2^ copies·g^−1^).

### Spatial distribution characteristics of nitrogen and phosphorus in sediments

#### Horizontal distribution characteristics of TN and TP

**Figure 4:**
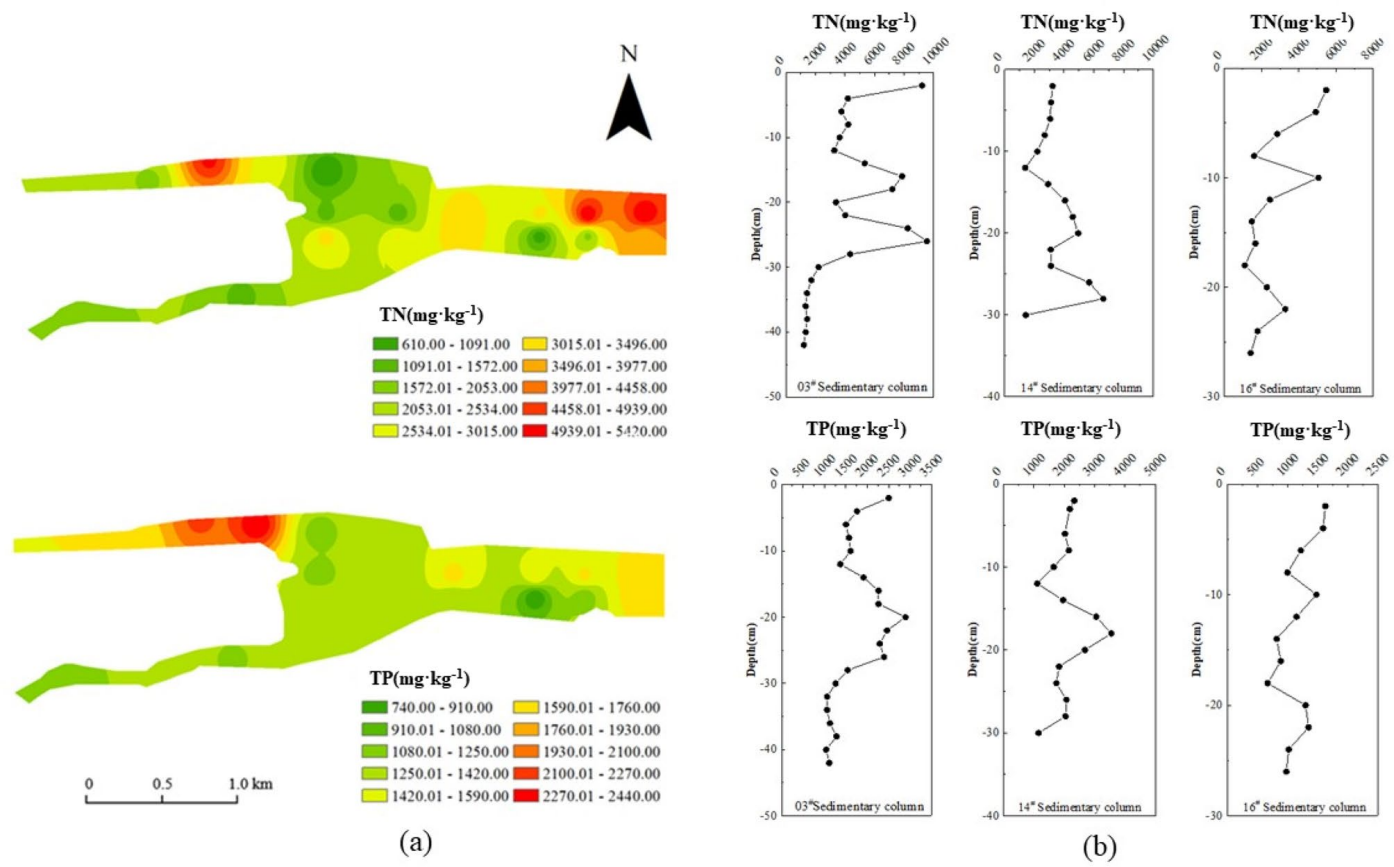
(**a**) Horizontal characteristics of TN and TP in the sediments. (**b**) Vertical characteristics of TN and TP in the sediments. (The figure was created by using ArcGIS software 10.2; Source:WGS 1984).

As shown in Fig. 4a, the TN content of the surface sediments (0–20 cm) from the Shahe Reservoir ranged from 610.00 to 5420.00 mg·kg^−1^, with an average value of 2759.44 ± 1450.54 mg·kg^−1^. The content of TN in the sediments from the point-source pollution area and downstream of the reservoir was significantly greater than that in the core area of ​​the reservoir, the river channel, and upstream of the reservoir. Shahe Reservoir is long and narrow. The reclaimed water (about 80,000 m^3^·d^−1^) flows into the Beisha reservoir (near the 13^#^sampling point), and the downstream of the reservoir is intercepted by a sluice dam. Therefore, flow velocity at the mouth and upstream of the reservoir is higher than at the middle and downstream. Although pollutants in the reservoir water body may have a tendency to gradually decrease from upstream to downstream, the particulate pollutants are more likely to be deposited in the downstream of the reservoir than at the entrance and upstream. Under normal water depth conditions, the particulate pollutants are at the mud–water interface and in deep water. While the self-purification rate of the area may not be high, the sedimentation is more obvious. Therefore, the phenomenon that pollutants in the sediments gradually accumulate from the upstream to the downstream of the reservoir is manifested in the TN content in the sediment, which increased sequentially from the upstream of the reservoir (1898.00 ± 1047.54 mg·kg^−1^) through the central area (2996.67 ± 1405.13 mg·kg^−1^) to the lower reaches (4500.00 ± 920.00 mg·kg^−1^). The highest value of TN in the sediments of the reservoir area was located at the 12^#^ sampling point (5420.00 mg·kg^−1^) downstream of the reservoir. The content of TN at sampling points 4^#^ and 5^#^ in the center area was relatively low (840.00 mg·kg^−1^, 1950.00 mg·kg^−1)^, due to the installation of aeration facilities in the overlying water body. Wu Bi^17^, Li Jinrong^18^, and others found that increased dissolved oxygen will promote the release of nitrogen and phosphorus nutrients from the sediments and thus reduce their content.

The content of TP in the surface sediments of Shahe Reservoir ranged from 740.00 to 2440.00 mg·kg^−1^, with an average value of 1444.33 ± 395.55 mg kg^−1^. As with the horizontal distribution of TN, TP also increased from the upper reaches of the reservoir (1264.00 ± 104.61 mg·kg^−1^) through the central area of ​​the reservoir (1340.00 ± 332.47 mg·kg^−1^) to the lower reaches of the reservoir (1750.00 ± 10.00 mg·kg^−1^). In the river course it was 1605.00 ± 522.61 mg·kg^−1^. Point-source pollution areas were slightly higher (2150.00 mg·kg^−1^) than the surface sediments of the reservoir area. This is because the point-source pollution area of ​​Shahe Reservoir is mostly near the urban–rural junction, with a high pollution-to-radius ratio.

The sources of pollutants are mostly domestic sewage, surface runoff, and pipeline sediments, and the proportion of phosphorus pollutants is often high. For example, Li Siyuan^19^ found that 11–30% NH_4_^+^-N, 18–35% TN, and 19–47% TP of the point-source pollution in the old city of Changzhou was from domestic sewage, while 23–46% NH_4_^+^-N, 43–56% TN, and 42–62% TP came from pipeline sediments.

After sewage and pipeline sediments entered Shahe Reservoir, the sediments and interstitial waters of the point-source pollution area showed significantly higher levels of nutrients such as nitrogen and phosphorus than were found in the river course, the upper reaches of the reservoir, the core area, and the lower reaches of the reservoir. This may be because the sediments of Shahe Reservoir are an important source of nitrogen and phosphorus nutrients. The content of TP in the surface sediments of the 16^#^ sampling point (1220.00 mg·kg^−1^) and 17^#^ sampling point (1100.00 mg·kg^−1^) of the Nansha River channel were low, due to the amount of algae and aquatic plants in the overlying water of the river. A large amount of phosphorus released by sediments is used by algae and aquatic plants^20^. At the same time, the river flow is faster, which reduces the phosphorus content in the sediments. The difference in the spatial distribution characteristics of TP in the surface sediments of Shahe Reservoir, in addition to hydraulic factors, may be related to the chemical environmental effects of different locations in the reservoir area^21^ and different microbial effects^22^.

#### Vertical distribution characteristics of TN and TP

Figure 4b shows the vertical change characteristics of TN and TP in the sediments of the Nansha River and Beisha River (near the point-source pollution area) and in the reservoir core area. The TN content of the 3# sediment column (0–42 cm) in the central area, the 14^#^ sediment column (0–30 cm) near the point-source pollution area, and the 16^#^ sediment column (0–26 cm) of the Nansha River channel ranged, respectively, between 1210.00 and 9540.00 mg·kg^−1^, 1400.00 and 6640.00 mg·kg^−1^, and 1100.00 and 5480.00 mg·kg^−1^. The mean values ​​were 4230.95 ± 2643.50 mg·kg^−1^, 3485.33 ± 1420.50 mg·kg^−1^, and 2723.08 ± 1456.81 mg·kg^−1^. For the same sampling points, the content range of TP ranged, respectively, between 1040.00 and 2890.00 mg·kg^−1^, 1110.00 and 3550.00 mg·kg^−1^, and 670.00 and 1630.00 mg·kg^−1^. The average values of TP were 1726.14 ± 561.22 mg·kg^−1^, 2100.67 ± 617.59 mg·kg^−1^, and 1161.54 ± 287.40 mg·kg^−1^.

The vertical distributions of TN and TP in the sediments showed a large change in the content of the surface layer and a small difference in the lower layer. From 10 cm, the content of TN and TP in the surface layer had an increasing trend. The distribution of TN and TP presented a three-stage feature of decrease-increase–decrease, with an enrichment layer at 10–20 cm. This may be due to the continuous increase of phosphorus load in the lake caused by human activity and industrial production in the upper reaches of Shahe Reservoir^23^. Zhang Wei et al.^12^ found that the water content of sediments below 30 cm in Shahe Reservoir was relatively stable and, based on the time of construction of the dam(1960), the sediment thickness was estimated to be about 30 cm, with a linear sediment deposition rate of 0.60 cm·yr^−1^. This is consistent with the analysis results of this study. It can be seen from Fig. 4b that the content of TN and TP below 30 cm in the 3^#^ column in the central area is in a stable state, and the 0–30 cm is mainly the sediment produced by external pollution since the construction of the reservoir.

### The relationship between nutrients and pathogens in sediments

**Figure 5:**
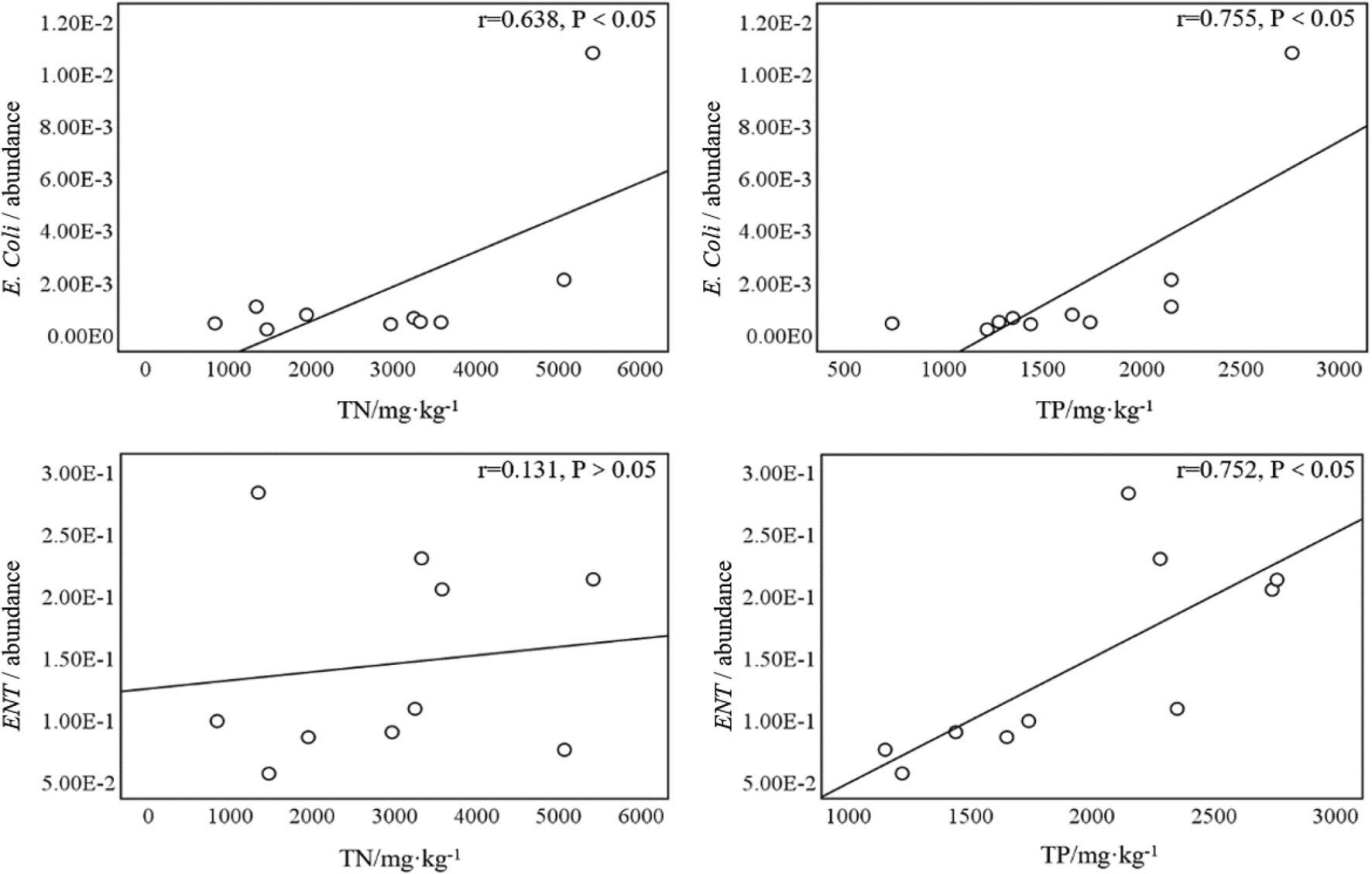
Pearson correlation analysis between pathogenic bacteria and TN and TP in the sediment. (The figure has been prepared using IBM SPSS 25.0 software).

The Pearson correlation analysis of the abundance of *E. coli* and *ENT* in the surface sediments and the horizontal distribution of TN and TP (Fig. 5) showed a significant positive correlation between *E. coli* and both TN (*r* = 0.638, *P* < 0.05) and TP (*r* = 0.755, *P* < 0.05); however, *ENT* and TN (*r* = 0.131, *P* > 0.05) were not significantly correlated, although there was a significant positive correlation with *ENT* and TP (*r* = 0.752, *P* < 0.05).

The Pearson correlation analysis of the relative abundance of *E. coli* and *ENT* in the sediments of Shahe Reservoir and the vertical distribution of TN and TP is shown in Table 2. There was a significant negative correlation between the *E. coli* in the 3^#^ sediment column in the center area and TN (*P* < 0.05) and also TP (*P* < 0.05); the *E. coli* in the 14^#^ sediment column had a significant negative correlation with TP (*P* < 0.05), but the correlation with TN was not significant (*P* > 0.05); in the 16^#^ sediment column, the correlations between *E. coli* and both TN and TP were not significant (*P* > 0.05).

**Table 2:** Pearson correlation analysis between pathogenic bacteria and TN and TP in the sediment.

Types of pathogens	Pearson correlation	3# Sedimentary column		14# Sedimentary column		16# Sedimentary column	
		TN	TP	TN	TP	TN	TP
*E. coli*	Correlation coefficient	− 0.940*	− 0.951*	0.499	− 0.945*	− 0.539	− 0.318
	Significance	0.018	0.013	0.392	0.015	0.349	0.602
*ENT*	Correlation coefficient	− 0.742	− 0.662	0.376	− 0.912*	− 0.981**	− 0.941*
	Significance	0.151	0.224	0.533	0.031	0.003	0.016

*At the 0.05 level (two-tailed), the correlation is significant.

**At the 0.01 level (two-tailed), the correlation is significant.

The nutrients in the water body will promote the growth and reproduction of aquatic plants and phytoplankton, and at the same time, phytoplankton will produce a large amount of organic matter through photosynthesis^24^. In addition, when the number of phytoplankton increases, the food intake of zooplankton also increases, and the excrement increases, which makes the organic matter in the water body increase. The growth and reproduction of aquatic plants also provides a suitable environment for the growth of microorganisms. Scholars such as Wang Mi pointed out in related investigations that TP and TN are environmental factors that affect phytoplankton in the North Canal; The study by Guo Feifei^25^ et al. on Hubei Jinshahe Reservoir showed that PO_4_^3−^-P affects the structure of microbial communities. Main environmental factors. Therefore, in addition to controlling microorganisms, the reservoir area should also strengthen the control of nutrients.

The correlation between *ENT* and the vertical distribution of TN and TP in the sediments of Shahe Reservoir was significantly different from that of *E. coli*. The main difference was that the correlations between *ENT* and TN and TP in the 3^#^ column of the center area were not significant; Beisha River channel *ENT* in the 14# sediment column had a significant negative correlation with TP (*P* < 0.05), but the correlation with TN was not significant (*P* > 0.05); in the Nansha River channel 16^#^ column, *ENT* had a significant negative correlation with TP (*P* < 0.05), and with TN a very significant negative correlation (*P* < 0.01).

It is worth noting that the Pearson correlation of the vertical distribution of *E. coli*, *ENT*, TN, and TP in the sediments of Shahe Reservoir was significantly different from correlation results of the horizontal distribution, mainly manifested in the significant negative correlations. The reason may be that changes in environmental conditions (pH more acid or alkali, higher water temperature, increased dissolved oxygen, stronger hydrodynamic conditions, etc.) release nutrients such as nitrogen and phosphorus^26^ and then, as a result, the content of TN and TP in the surface layer of the sediment becomes higher and the content in the deep layer becomes lower. The migration and fate conditions of *E. coli* and *ENT* are different from those of nitrogen and phosphorus, as they are mainly affected by factors such as strain type, bacterial solution concentration, ionic strength, ion type, median particle size, pore flow rate, etc^27^. As a result, the Pearson correlation between *E. coli* and *ENT* in the sediments of Shahe Reservoir and the vertical distribution of TN and TP showed a negative correlation.

## Conclusions

1. The horizontal distribution characteristics of *E. coli* in the sediments of Shahe Reservoir can be mainly expressed as: downstream of the reservoir > point-source pollution area > upstream of the reservoir > center area of ​​the reservoir > river course. The horizontal distribution characteristics of *ENT* in the sediments of Shahe Reservoir can be summarized as: center area of ​​the reservoir > point-source pollution area > upstream and downstream of the reservoir > river course. The vertical distribution characteristics of *E. coli* and *Enterococcus* in the sediments both showed a trend of increasing with the increase of depth, and both peaked at 15–25 cm.
2. The horizontal distributions of TN and TP in the sediments of Shahe Reservoir were highest at the point-source pollution area. The middle and lower reaches of the reservoir were higher than the river channel, the upper reaches of the reservoir, and the center of the reservoir. There was a characteristic of gradual accumulation from top to bottom along the reservoir. The vertical distribution of TN and TP was characterized by a three-stage distribution of decrease–increase–decrease, and there was an enrichment layer at 10–20 cm.
3. Pearson correlation analysis showed that, in the horizontal distribution of Shahe Reservoir sediments, *E. coli* had a significant positive correlation with total nitrogen (*P* < 0.05) and total phosphorus (*P* < 0.05); there was no correlation between *ENT* and total nitrogen, although there was a significant positive correlation between *ENT* and total phosphorus (*P* < 0.05). In terms of vertical distribution, *E. coli* had a significant negative correlation with total nitrogen (*P* < 0.05) and total phosphorus (*P* < 0.05) in the central area of ​​the reservoir, while *ENT* had a significant negative correlation with total nitrogen (*P* < 0.01) near the point-source pollution area and a significant negative correlation with total phosphorus (*P* < 0.05).
4. The good correlation between nutrients and pathogens in the sediments can provide a theoretical basis for controlling the nutrients in lakes and reservoirs to control pathogen pollution in the future.
